# Alterations of Left Ventricular Function Persisting during Post-Acute COVID-19 in Subjects without Previously Diagnosed Cardiovascular Pathology

**DOI:** 10.3390/jpm11030225

**Published:** 2021-03-22

**Authors:** Mariana Tudoran, Cristina Tudoran, Voichita Elena Lazureanu, Adelina Raluca Marinescu, Gheorghe Nicusor Pop, Alexandru Silvius Pescariu, Alexandra Enache, Talida Georgiana Cut

**Affiliations:** 1 Department VII, Internal Medicine II, Discipline of Cardiology, University of Medicine and Pharmacy “Victor Babes” Timisoara, E. Murgu Square, Nr. 2, 300041 Timisoara, Romania; tudoran.mariana@umft.ro; 2 Center of Molecular Research in Nephrology and Vascular Disease, Faculty of Medicine, University of Medicine and Pharmacy "Victor Babes" Timisoara, E. Murgu Square, Nr. 2, 300041, Timisoara, Romania; 3 County Emergency Hospital Timisoara, 300041 Timisoara, Romania; 4 Department XIII, Discipline of Infectious Diseases, University of Medicine and Pharmacy “Victor Babes” Timisoara, E. Murgu Square, Nr. 2, 300041 Timisoara, Romania; vlazureanu@gmail.com (V.E.L.); Marinescu_adelina24@yahoo.com (A.R.M.); Talida.cut@gmnail.com (T.G.C.); 5 Department VI, Cardiology, University of Medicine and Pharmacy “Victor Babes” Timisoara, E. Murgu Square, Nr. 2, 300041 Timisoara, Romania; pop.nicusor@umft.ro (G.N.P.); pescariu.alexandru@umft.ro (A.S.P.); 6 Department VIII, Discipline of Forensic Medicine, University of Medicine and Pharmacy “Victor Babes” Timisoara, E. Murgu Square, Nr. 2, 300041 Timisoara, Romania; enache.alexandra@umft.ro

**Keywords:** COVID-19, heart failure, inflammation, cytokine storm, transthoracic echocardio-graphy, left ventricular systolic dysfunction, diastolic dysfunction

## Abstract

(1) Background: Coronavirus infection (Covid-19) has emerged as a severe medical condition, associated with high pulmonary morbidity and often with cardiovascular (CV) complications. This study aims to evidence the persistence of left ventricular (LV) systolic function (LV-SF) alterations and diastolic dysfunction (DD) in COVID-19 patients without history of cardiovascular (CV) diseases by transthoracic echocardiography (TTE). (2) Methods: 125 patients, aged under 55 years, hospitalized during the first outbreak of Covid-19 for moderate pneumonia, underwent a comprehensive cardiologic examination and TTE at 6–10 weeks after discharge. Their initial in-hospital laboratory data and thorax computer tomography (TCT) were accessed from the electronic database of the hospital. (3) Results: with TTE, we documented alterations of LV-SF and DD in 8.8% of patients and in 16.8% only patterns of DD, statistically correlated with the initial levels of creatin-kinase (CK-MB) and inflammatory factors. Multivariate regression analysis evidenced that CK-MB levels, age, and body mass index (BMI) are responsible for 65% of LV-SF decrease. (4) Conclusions: Alterations of LV-SF and DD are frequent in post-acute COVID-19 infection and are responsible for the persistence of symptoms. Elevated myocardial necrosis markers during the acute phase seem to predict subsequent alteration of cardiac performance.

## 1. Introduction

COVID-19, determined by the infection with Coronavirus (SARS-CoV-2), generated a global pandemic, associated with significant morbidity and mortality [1,2,3,4]. Although respiratory pathology prevails, COVID-19 determines multiple organ injuries, including cardiovascular (CV) complications, frequently responsible for a fatal outcome [5,6,7], especially in elderly patients with underlying CV diseases. The spectrum of CV complications is very large, ranging from sinus tachycardia or hypotension to various arrhythmias, myocardial ischemia, acute myocarditis with heart failure (HF), or even pulmonary thromboembolism (TEP) [8,9] and right ventricular dysfunction (RVD) [7,10,11]. The suspected pathophysiological mechanisms responsible for the CV impairment are multiple: direct injury of cardiomyocytes by the virus, multiple inflammatory responses triggered by a cytokines storm, endothelial dysfunction or even vasculitis, destabilization of existing coronary plaques, TEP, or microthrombogenesis, often aggravated by hypoxemia [11,12,13]. In many hospitalized COVID-19 patients, the assessment of biomarkers indicating myocardial injury (high-sensitivity cardiac troponin I, creatin-kinase—CK-MB) revealed levels above the upper limit of the reference range [2,14] suggesting acute myocarditis, often accompanied by impaired left ventricular (LV) systolic function (LV-SF) and ischemia [15]. Although transthoracic echocardiography (TTE) offers the possibility of an accurate evaluation of the systolic and diastolic function, in most cases, due to the increased risk of contamination and shortage of qualified personnel, a comprehensive transthoracic TTE is seldom performed, being reserved for selected cases [16]. 

Since COVID-19 is a recent disease, the evolution of CV abnormalities has not yet been established, but as we are confronted with its second or even third outbreak, we will be facing new challenges regarding the long-term consequences of this disease. It is known that many patients experience symptoms like fatigue, reduced effort capacity, dyspnea, palpitations, or chest discomfort a long time after COVID-19, and routine cardiologic exams and TTE often reveal altered myocardial contractility and impaired cardiac performance. Terms as post-acute COVID include the persistence of symptoms from 3 to 12 weeks after the acute phase, and long COVID describes the presence of manifestations even after 12 weeks [17].

This study aims to explore, by TTE, the alterations of the LV systolic and diastolic function in patients not yet recovered from COVID-19, at 6–10 weeks after their discharge from the hospital. Another aim is to document if the modified TTE parameters, indicating alterations of LV-SF or diastolic dysfunction (DD), are related to the magnitude of the initial immune response and levels of CK-MB, or the severity of the primary pulmonary injury, assessed on thorax computer-tomography (TCT).

## 2. Materials and Methods

### 2.1. Study Population

Study population: By analyzing the electronic database of the Hospital for Infectious Diseases from Timisoara of all 768 patients hospitalized for a SARS-CoV2 infection during the first COVID-19 outbreak (28 February and 31 July 2020) when hospitalization was mandatory for all individuals infected with SARS-CoV 2 virus, we observed that 69 patients died and 699 were discharged alive after a median in-hospital stay of 9.8 (8.3 to 12) days. Of these, 415 patients were classified according to the WHO’s criteria as mild/moderate forms of COVID-19 pneumonia [4]. Of these, only 254 patients younger than 55 years without a history of significant associated CV pathology were considered to be suitable for our study. Patients older than 55 years were not included because DD, due to increased CV stiffness, is not uncommon in this category. Within less than 10 weeks after discharge, these potential subjects were contacted by phone and invited to attend a cardiologic exam with TTE. Of those invited, 182 patients agreed to undergo this assessment and were attentively evaluated by a cardiologist to identify an underlying CV pathology. Fifty-seven participants were diagnosed with associated medical conditions and were therefore not considered adequate to take part in our investigation. Before the collection of any data, all 125 remaining patients signed an individual informed consent form and were included in this study according to the criteria outlined in Table 1. Their baseline clinical characteristics, TTE, TCT, and laboratory data, as well as their therapy, were obtained retrospectively, by reviewing their electronic medical records. 

The Local Scientific Research Ethics Committee of our hospital approved the design and methodology of our study (No. 209/17 October 2020).

### 2.2. Methods

Thorax computer tomography: based on the TCT results, all patients with pneumonia were classified at admission in the hospital in mild (<30% pulmonary injury) or moderate (30–60% lesions) forms. In the context of our study, TCT images were re-examined and pulmonary lesions were semi-quantitatively classified, according to the scoring proposed by Francone et al. in their study [18]. Taking into account the extent of the parenchymal damage, scores for each of the 5 lung lobes were calculated (0: no injury; 1: <5% lesion; 2: 5–25% lesions; 3: 26–50% damage; 4: 51–75% injury and 5: >75% involvement). The sum represents the global TCT score (0 to 25), the higher the score, the more severe the lung damage.

Echocardiographic examination: to avoid inter-observer differences, the same experienced operator performed all TTE measurements, according to guidelines recommendations [19]. After a regular exam of the cardiac morphology and function, the following evaluations were performed:

Left ventricular (LV) systolic dysfunction was assessed from apical 4-chamber view and included:

- the assessment of LV ejection fraction (LVEF); by the modified Simpson rule, values of LVEF < 50% being considered abnormal;
- the measurement of the lateral mitral annular plane systolic excursion (MAPSE); values < 10 mm being considered pathologically;
- the assessment, in Tissue Doppler imaging (TDI), of the average systolic mitral annular velocity (LV-S’); values under 0.07 m/s being considered abnormal;
- the quantification of the LV global longitudinal strain (LV-GLS) was realized from apical 2-, 3-, and 4-chamber view, the region of interest being automatically generated and, after tracing the LV endocardial border, manual corrections were subsequently performed to fit the thickness of the LV myocardial wall [20,21,22]. Values under −18% suggested an impaired LV systolic function (LV-SF).

Left ventricular diastolic dysfunction (DD) comprised:

- the assessment of left ventricular mass index (LVMI); values of over 115 g/m2 for males and 95 g/m2 for females defined left ventricular hypertrophy (LVH);
- left atrial volume index (LAVI); values over 34 mL were considered pathological;
- in pulsed Doppler, in apical 4-chamber view, we registered the mitral inflow at the level of the mitral valve annulus and analyzed the peak early diastolic velocity (E), the late diastolic velocity (A), and the E/A ratio.
- TDI was used to record the early diastolic velocity (e’) and the late diastolic velocity at the level of the septal and lateral mitral annulus. Subsequently, an average and E/e’ ratio were calculated. Type I of DD was defined by an E/A ratio ≤0.8 and E < 50cm/Sec, while type III DD was confirmed by an E/A ratio of over 2. In case of an E/A ratio ≤ 0.8, but with an E of over 50 cm/Sec, or if the E/A was between 0.8 and 2, a type II DD was considered, and was certified if at least two of the following three criteria were present: an average E/e’ > 14, LAVI > 34 mL/m2, and/or TRV > 2.8 m/Sec. In cases where only one of the three previously mentioned criteria were fulfilled, a type I DD was diagnosed [23].

Right ventricular (RV) performance was evaluated in 4-chamber view, visualizing the entire RV:

- tricuspid annular plane systolic excursion (TAPSE) was measured at the level of the lateral tricuspid valve annulus in M-Mode;
- tricuspid regurgitation velocity (TRV) was recorded by continuous-wave Doppler, from the apical window, at the level of the tricuspid valve;
- echocardiographically determined systolic PAP (sPAP) was assessed based on the peak TRV, taking into account the right atrial pressure, determined by measuring the inferior vena cava diameter and its respiratory variations. In this study, we considered that sPAP values of ≥35 mmHg at rest indicate PH [14,16] with severity ranging from mild (35–44 mmHg) to moderate (45–60 mmHg) to severe (>60 mmHg) [24,25];
- RV global longitudinal strain (RV-GLS) was performed in apical 4 chamber view [25,26] and, according to the latest international recommendations, RV dysfunction (RVD) was certified by either TAPSE < 17 mm and/or RV-GLS < −28% [26].

A first endpoint of our study was to evaluate, in terms of cardiovascular complications, the outcome of 125 patients in whom we ruled out by history, clinical examination, and TTE a pre-existing cardiovascular condition that would have explained the presence of cardiac alterations assessed by TTE.

The second endpoint was to identify the patients with alterations of the cardiac performance to refer them for further evaluation and to monitor their evolution under therapy. 

Statistical analysis: The Statistical Package for the Social Sciences v.25 (SPSS, Chicago, IL, USA) was employed to perform data analysis. We presented continuous variables as mean and standard deviation (SD) or median and interquartile range (IQR), and categorical variables as frequency and percentages. Because the results of the normality test (Shapiro–Wilk) showed a non-Gaussian distribution, we continued the analysis by using nonparametric tests. For comparing the three groups (A, B, C), we employed the Kruskal–Wallis test, followed by post-hoc analysis with Mann–Whitney U test and Bonferroni correction for pairwise comparisons. To compare TTE parameters, the Mann–Whitney U test was employed. Fisher’s exact test was employed to evaluate the significance of differences in the proportions of nominal variables. We employed Spearman’s correlation test to assess the potential connection between LV-GLS and E/e’ ratio with other TTE parameters and laboratory results. We built several multivariate regression models to assess the individual impact of several confounding factors on the variance of continuous variables, and the model was validated based on the accuracy of prediction and R squared. In the final regression equations, the predictors were accepted according to a repeated backward-stepwise algorithm (inclusion criteria *p* < 0.05, exclusion criteria *p* > 0.10) to obtain the most appropriate theoretical model to fit the collected data. We considered that *p* values under 0.05 indicate statistically significant differences.

## 3. Results

Our study included 125 patients, 63 women and 62 men, with ages between 29 and 55 years, median age 47 (40–51.5). All had a recent hospitalization for a mild/moderate SARS-CoV-2 pneumonia, confirmed by RT-PCR and TCT during the first outbreak of COVID-19 less than 10 weeks before. None of them had severe respiratory insufficiency requiring mechanical ventilation or ICU stay. Of those patients, 29.6% were overweight and 28.8% had obesity. When they were contacted by phone, 57 of them (53.6%) still reported symptoms, most frequently fatigue, dyspnea, and palpitations. After the inclusion in the study, patients’ laboratory data were accessed from the electronic database of the hospital, and their TCTs were re-analyzed and a global TCT COVID score calculated. All these results are displayed in Table 2. The statistical analysis revealed no significant differences regarding gender, age, and BMI between patients with systolic dysfunction or DD and those with normal cardiac performance, but there were considerable ones regarding the severity of the initial lung injury, assessed on TCT, levels of inflammatory factors, and CK-MB between groups. 

Therapy during hospitalization was prescribed according to current recommendations with protease inhibitors (remdesivir (Gilead Sciences, Foster City, CA, USA), lopinavir/ritonavir-AbbVie, Ludwigshafen, Germany), antibiotics (cefuroxime (Antibiotice, Iasi, Romania) or azithromycine-Sandoz, Targu-Mures, Romania), corticoids (dexametazone-Medochemie Ltd., Limassol, Cyprus), and anticoagulants (apixaban (Bristol-Myers Squibb/Phizer EEIG, Dublin, Ireland) or rivaroxaban-Bayer AG, Leverkusen, Germany), continued 40 days after discharge in a prophylactic dose.

At the inclusion in the study, at 6–10 weeks after the discharge from the hospital, a detailed clinical exam and a comprehensive TTE were performed in all patients that should have recovered from COVID-19 at that time. Values are presented in Table 3, and statistically significant differences between patients with normal cardiac performance and those with DD associated or not with altered LV-SF were noted. Except for mentions of trivial mitral or aortic regurgitations, all subjects had an initially normal TTE, performed either before the admission, in the emergency room, or during the first days of hospitalization, but it was a minimal exam, mentioning only that there were no significant cardiac abnormalities, without detailed measurements of all studied parameters.

Referring to our study group, in 93 patients (74.4%), TTE parameters characterizing the LV systolic and diastolic function were within normal limits, although we identified in 7 individuals (4 men and 3 women) mild LVH and another 4 patients had borderline values of RV-GLS (Table 3). It is to mention that, referring to the whole study group, as expected, we highlighted statistically significant correlations between TTE parameters characterizing LV function like LV-GLS with MAPSE, LV-EF, and VS’ average (r = −073, r = −0.79 and respectively r = −0.6, all of them with *p* < 0.001). 

In a subset of 11 patients (8.8%), we identified alterations of LV-SF, certified by LV-GLS < 17, MAPSE < 10, and LVEF under 50 (between 36 and 48%). All of them had associated DD: 6 of type III, with E/A > 2 and 5 of type II. LVH was evidenced in 4 men and 1 woman. Other associated findings, detected in all these patients, were RVD and PH (of mild or moderate severity).

In another subgroup of 21 patients (16.8%), 13 women and 8 men, with normal LV-SF, we documented the presence of DD: 6 subjects had DD of type I and 15 of type II. Ten patients (6 women and 4 men) had associated LVH, and 7 of them had mild RVD. Taking into account that all 11 patients with altered LV-SF had associated DD, the real prevalence of this dysfunction rises to 25.6%.

When we examined the existence of correspondences between LV-GLS with other TTE parameters characterizing cardiac performance in all patients with altered LV-SF and/or DD, we documented, by using Spearman’s correlation, statistically significant correlations between all these TTE parameters, as well as between LV-GLS with TCT scores and laboratory data assessed at the admission in the hospital, Table 4.

Referring to LV-GLS as the most sensitive parameter characterizing the LV function, if we take into account only the 11 patients with impaired LV-SF, the statistical analysis by using Spearman’s correlation, reveal statistically significant correlations with several inflammatory markers like Interleukin-6, CRP, and with CK-MB (r = 0.88, r = 0.829, and r = 0.72, with *p* < 0.001, *p* = 0.002, and *p* = 0.012, respectively) and moderate ones with age, BMI, and TCT score (r = 0.69, r = 0.53, and r = 0.59 with *p* = 0.017, *p* = 0.09, and *p* = 0.05, respectively). A strong correlation with MAPSE (0.85, *p* = 0.001) was also documented, as well as moderate ones with LVEF, RV-GLS, and TRV (r = −0.55, r = 0.61 and r = 0.64). 

Considering that elevated values of E/e’ ratio were the most common finding in patients with DD, by using the Spearman’s correlation, the statistical analysis evidenced statistically significant correlation of the E/e’ ratio with age and BMI (r = 0.81 and r = 0.67, *p* < 0.001), with COVID TCT score (r = 0.79, *p* < 0.001), and with markers of inflammation determined at the admission in the hospital: CRP, interleukin-6, and fibrinogen (r = 0.9 with *p* < 0.001, respectively r = 0.63 with *p* = 0.002). We evidenced moderate correlations with other parameters characterizing DD: LAVI (r = 0.77, *p* < 0.001), TRV (r = 0.47, *p* = 0.029), and the number of days until negativation of PCR (r = 0.7 *p* < 0.001). Although LVMI should be a factor that strongly influences DD, due to different limits for men and women, the correlation was not statistically significant.

We employed in our patient group a multivariate linear regression analysis to determine the most significant independent predictor factors that could explain the occurrence of altered LV-GLS associated with DD or not. The forward stepwise method was used to build the regression model, and the best model was appreciated based on Akaike information criteria (AIC). After the adjustment for all other TTE parameters characterizing the cardiac performance as potential confounding factors, we identified that the initial values of CK-MB, more advanced age, and BMI are responsible for 65% of the reduced values of LV-GLS (R2 = 0.649, β = 0.128, S.E. = 0.009, *p* < 0.001). For each increase of CK-MB with 1 IU/L, the risk of a reduced LV-SF augments by 1.3%. Similar results were obtained also for the E/e’ ratio.

Patients with cardiac impairment were included in a monitoring program and recommended to undergo cardiac magnetic resonance imaging (MRI), and therapy with angiotensin converting enzyme (ACE) inhibitors and antiischemic agents (perindopril, respectively trimetasidine MR-Les Laboratoires Servier, Suresnes cedex, Paris, France) and in several cases beta-blockers (nebivolol-Berlin Chemie, Berlin, Germany) was initiated.

## 4. Discussion

The increased prevalence of CV complications in the acute phase of COVID-19 is a debated topic in numerous scientific articles [27,28,29]. Most of these studies were performed during the acute phase of COVID-19, in hospitalized patients, regardless of their age or associated diseases, independent of the severity of the disease, including cases assisted in intensive care units with an increased rate of acute respiratory distress syndrome and/or pulmonary thromboembolism, requiring special therapeutic measures [30,31]. There is a consensus that RVD and PH prevail, but authors are debating over evidence of heart failure (HF) with reduced (HFrEF) of preserved EF (HFpEF) after SARS-CoV2 infection [27]. For example, in their study, Szekeley et al. sustain that RVD was observed in 39% of patients, followed by DD (in 16%) and reduced LV-SF in 10%, but there are no details about the associated diseases, especially CV pathology [28]. In our study, performed at 6–10 weeks after discharge from the hospital, in younger patients, without previous significant CV disease, the prevalence of RVD was only 14.4%. We evidenced in 8.8% of subjects altered LV-SF associated with DD, and in other 16.8%, only DD with normal systolic performance, similar to data reported in the literature [27,28]. It is possible that we documented a higher prevalence of altered cardiac performance in patients with moderate forms because mostly symptomatic patients, more than half of them suffering from post-acute COVID, agreed to take part in our study. It is to mention that 74.4% of our subjects had no abnormalities of the cardiac performance at this evaluation performed after recovery from COVID-19. However, a limitation of our study is that we do not have an accurate TTE assessment of all studied parameters at baseline because the echocardiographic exam performed at the admission in the hospital was only a basic one.

The pathogeny of DD and HFpEF in COVID-19 patients represents a controversial subject, with several hypothesis being proposed: a direct viral infiltration of the myocardium, inflammation, ischemia, or subsequent fibrosis. The exacerbation of a preexisting DD or the unmasking of subclinical forms in subjects with risk factors cannot be ruled out [27]. By selecting in our study only younger individuals, we tried to limit the impact of age, a well-known factor responsible for alterations of cardiac performance, especially in DD, but we had an increased prevalence of obesity (28.8%), and 29.6% of our subjects were overweight, an aspect that can explain, at least partially, the increased prevalence of DD. The echocardiographic classification of DD according to guidelines recommendations [23] was somewhat challenging because, in several patients, the elevated TRV could be attributed to the pulmonary injury, and increased LAVI could represent the consequence of altered LV-SF in these subjects. That is why we employed the E/e’ ratio for the statistical analyses. However, it is noteworthy that statistically significant correlations (with *p* < 0.001) were evidenced between TTE parameters characterizing LV-SF, as well as DD, and the initial levels of CK-MB and several inflammatory biomarkers (CRP, interleukin-6, fibrinogen) supporting the hypothesis that the cytokine storm, following the acute viral injury, determines myocardial lesions that could potentially progress to fibrosis affecting the cardiac performance [13].

Even more concern has arisen in the last period, regarding the persistence of symptoms like dyspnea, fatigue, reduced effort capacity, in COVID-19 patients, a long time after the acute disease, when they should have totally recovered. Different terms and pathogenies have been proposed, but precise information is still missing. Few studies are referring to the evolution of CV complications in patients who had suffered a SARS-Cov-2 infection, but still have symptoms starting from 3 to 12 weeks after the acute phase, the so-called post-acute COVID-19 and even less in those who have not yet achieved their base-line health after 12 weeks, considered to suffer from the Long COVID syndrome [17,32]. However, before considering all these symptoms as being functional ones, a rigorous cardiologic exam with TTE is necessary to rule out CV complications associated with SARS-CoV-2 infection. 

## 5. Conclusions

Alterations of cardiac performance are not so uncommon after COVID-19 and could explain, at least partially, the persistence of symptoms in the post-acute COVID-19 phase. Elevated myocardial necrosis markers and inflammation during the acute infection, older age, and increased body mass index seem to predict subsequent impairment of the systolic or diastolic cardiac function.

## Figures and Tables

**Table 1:** Inclusion and exclusion criteria.

Inclusion Criteria	Exclusion Criteria
-aged under 55 years	-age over 55 years
-patients hospitalized during the first COVID-19 outbreak for a mild/moderate form	-severe/critical form of COVID-19 pulmonary infection
-diagnosis confirmed by a positive result of real-time reverse transcriptase–polymerase chain reaction of nasal and pharyngeal swabs	-respiratory insufficiency, requiring mechanical ventilation and/or intensive care unit (ICU) stay, during hospitalization
-TCT documented pneumonia	-absence of TCT or of pulmonary lesions on TCT
-a normal routine TTE performed at admission	-patients diagnosed during the study with significant cardiac pathology
-without history of significant CV diseases or diabetes mellitus	

**Table 2:** Demographic and laboratory characteristics of study population.

Characteristics	A—Patients without DD and Normal LV-SF *n* = 93 (74.4%)	B—Patients with DD and Normal LV-SF *n* = 21 (16.8%)	C—Patients with Altered LV-SF *n* = 11 (8.8%)	*p*
Gender: Male Female	46 (49.46%) 47 (50.53%)	8 (38.08%)13 (61.9%)	8 (72.72%) 3 (27.27%)	0.176 ^a^
Age (median)	46 (39.5–50)	50 (43.5–54)	49 (42–52)	0.239 ^b^
BMI (Kg/m^2^)	26.7 (23.5–30.3)	28.56 (26.84–31.18)	29.4 (24.47–31.8)	0.106 ^b^
TCT global score	4 (3–5)	7 (5–8)	10 (8–12)	<0.001 ^b^
<10 points ≥10 points	91 (97.84%) 2 (2.15%)	12 (57.14%) 9 (42.85%)	5 (45.45%) 6 (54.54%)	<0.001 ^a^
Laboratory results				
Leukocytes (/uL)	5761 (4279–7604.5)	6970 (4995–9985)	5820 (3580–8880)	0.255 ^b^
Lymphocytes (/uL)	1790 (1410–2454.5)	2030 (1415–3035)	1500 (890–2230)	0.166 ^b^
D-dimer (ng/mL)	0.33 (0.30–0.41)	0.4 (0.33–0.48)	0.3 (0.25–0.35)	0.026 ^b^
CRP (mg/L)	28.05 (8.76–39.95)	35.48 (21.9–50)	57.7 (36.7–59.78)	<0.001 ^b^
Fibrinogen (g/L)	2.92 (2.51–3.54)	4.12 (3.22–5.4)	5.45 (4.58–6.57)	<0.001 ^b^
Interleukin-6 (pg/mL)	5.1 (3.65–6.9)	6.9 (5.56–9.3)	10.6 (8.6–12)	<0.001 ^b^
CK-MB (UI/L)	25 (20–28)	30 (22.5–42.5)	60 (56–70)	<0.001 ^b^
Creatinine (mg/dL)	0.78 (074–0.86)	0.83 (0.74–0.98)	0.97 (0.88–1.05)	<0.001 ^b^
Time to normal RT-PCR (days)	16 (14–17)	15 (14–20)	20 (14–27)	0.025 ^b^

**Legend:** DD—diastolic dysfunction. LV-SF—left ventricular systolic function. BMI—Body mass index; TCT—Thorax computer tomography; CRP—C reactive protein. CK-MB—creatin-kinase MB. PCR—polymerase chain reaction; ^a^ Counts (frequency), Fisher-exact test; ^b^ median [IQR], ^a^ Fisher-exact test; ^b^ Kruskall–Wallis test.

**Table 3:** Transthoracic echocardiography (TTE) parameters determined in the study group.

Characteristics	Patients without DD and Normal LV-SF *n* = 93 (74.4%)	Patients with DD with and without Altered LV-SF *n* = 32 (25.6%)	*p*
Echocardiography parameters characterizing left ventricular function			
LAVI (ml/m^2^)	20.6 (19.55–24.9)	34.2 (24.4–35)	<0.001
LVMI (g/m^2^)	96.7 (91.5–107.05)	104.3 (94.7–114.5)	0.008
MAPSE lateral (mm)	18 (15.5–21)	12.5 (8.25–19.75)	<0.001
LVEF Simpson (%)	60 (56–65)	52 (45–58.75)	<0.001
VE (m/s)	0.67 (0.63–0.72)	0.7 (0.54–0.81)	0.356
VA (m/s)	0.6 (0.55–0.67)	0.63 (0.44–0.75)	0.939
E/A	1.1 (1.02–1.15)	1.03 (0.75–1.75)	0.523
Ve’ average (cm/s)	7.5 (6.7–8.1)	5.4 (4.7–5.9)	<0.001
E/e’ average	8.93 (8.52–9.51)	14.2 (10.84–14.51)	<0.001
VS’ average (cm/s)	9 (8.3–9.6)	8.3 (6.2–9.4)	0.007
LV-GLS (%)	−20 (−22–−19)	−18 (−20–−15.2)	<0.001
Echocardiography parameters for right ventricular function			
TAPSE lateral (mm)	23 (22–24)	21 (17.8–23)	<0.001
TRVmax (m/s)	2.3 (2.1–2.46)	3 (2.68–3.23)	<0.001
sPAP (mmHg)	26.16 (22.64–29.2)	41 (33.8–46.9)	<0.001
RV-GLS (%)	−29 (–30–−28)	−27 (–28–−19)	<0.001

**Legend:** DD—diastolic dysfunction; LV-SF—left ventricular systolic function; LAVI—left atrial volume index; LVMI—left ventricular mass index; MAPSE—mitral annular plane systolic excursion; LVEF—left ventricular ejection fraction; VE-velocity of the peak early mitral inflow wave(E); VA—velocity of the late mitral inflow wave(A); E/A—peak mitral inflow early (E) to late (A) diastolic velocities in pulsed Doppler; E/e’—early mitral inflow diastolic velocity E to average e′ velocity (E/e’) in pulsed tissue Doppler; VS’-average systolic velocity in pulsed tissue Doppler; LV-GLS—left ventricular global longitudinal strain; TAPSE—tricuspid annular plane systolic excursion; TRVmax—peak tricuspid regurgitation velocity; sPAP—systolic pressure in the pulmonary artery; RV-GLS—right ventricular global longitudinal strain; Mann–Whitney U test.

**Table 4:** Correlations between left ventricular global longitudinal strain (LV-GLS), other TTE parameters, and laboratory data in patients with diastolic dysfunction (DD) coinciding or not with altered left ventricular systolic function (LV-SF).

LV−GLS	Age	LVEF Simpson	MAPSE	TRVMax	RV−GLS	E/e’	TCT Score	CK−MB	CRP	Inter-Leukin6	Fibri-Nogen
R	0.272	−0.797	−0.734	0.507	0.290	0.265	0.367	0.631	0.390	0.404	0.312
p	0.002	0.000	0.000	0.000	0.001	0.003	0.000	0.000	0.000	0.000	0.000
95%CI	0.093	−0.864	−0.828	0.343	0.108	0.080	0.182	0.494	0.220	0.234	0.129
	0.416	−0.706	−0.622	0.640	0.467	0.452	0.540	0.737	0.560	0.556	0.494

**Legend:** LV-SF—left ventricular systolic function; TTE—transthoracic echocardiography; DD—diastolic dysfunction; LVEF—left ventricular ejection fraction; MAPSE—mitral annular plane systolic excursion; TRVmax—peak tricuspid regurgitation velocity; RV-GLS—right ventricular global longitudinal strain; E/e’—early mitral inflow diastolic velocity E to average e′ velocity in pulsed tissue Doppler; TCT—thorax computer tomography. CK-MB—creatin-kinase MB. CRP—C reactive protein. Spearman correlation test.

## Data Availability

The data are available on https://preventim.ro/public/pages/2.php.
